# Supplementary material for: Upcycled high-strength aluminum alloys from scrap through solid-phase alloying
Source: Nat Commun. 2024 Dec 10;15:10664. doi: 10.1038/s41467-024-53062-2 (PMC11631979; doi:10.1038/s41467-024-53062-2)
Supplement: Supplementary file 1 — Supplementary Information [file 41467_2024_53062_MOESM1_ESM.pdf]

Supplementary information

Upcycled High-Strength Aluminum Alloys from Scrap through Solid-Phase Alloying

Tianhao Wang [1], Xiao Li [1 *], Zehao Li [1], Tingkun Liu [1], Xiang Wang [1],
Arun Devaraj [1], Cindy A Powell [1], Jorge F dos Santos [1 *]

[1]Pacific Northwest National Laboratory, Richland, WA 99354, USA
*Corresponding author:
Jorge F dos Santos; jorge.dossantos@pnnl.gov; (509)-372-6261
Xiao Li; xiao.li@pnnl.gov; (509) 375-6897

Supplementary Note 1 Microstructural comparison between recycled and upcycled specimens

The cross and longitudinal sections of the extruded rods are displayed in Fig. 1 using optical microscopy and scanning electron microscopy (SEM) images. The chemical composition variation between recycled and upcycled rods is compared using the SEM and energy dispersive spectroscopy (EDS) map analysis, which was conducted on the half radius (R) of the cross-sections. There is an evident content increase in Mg, Zn, and Cu in the upcycled specimen as compared with the recycled specimen.

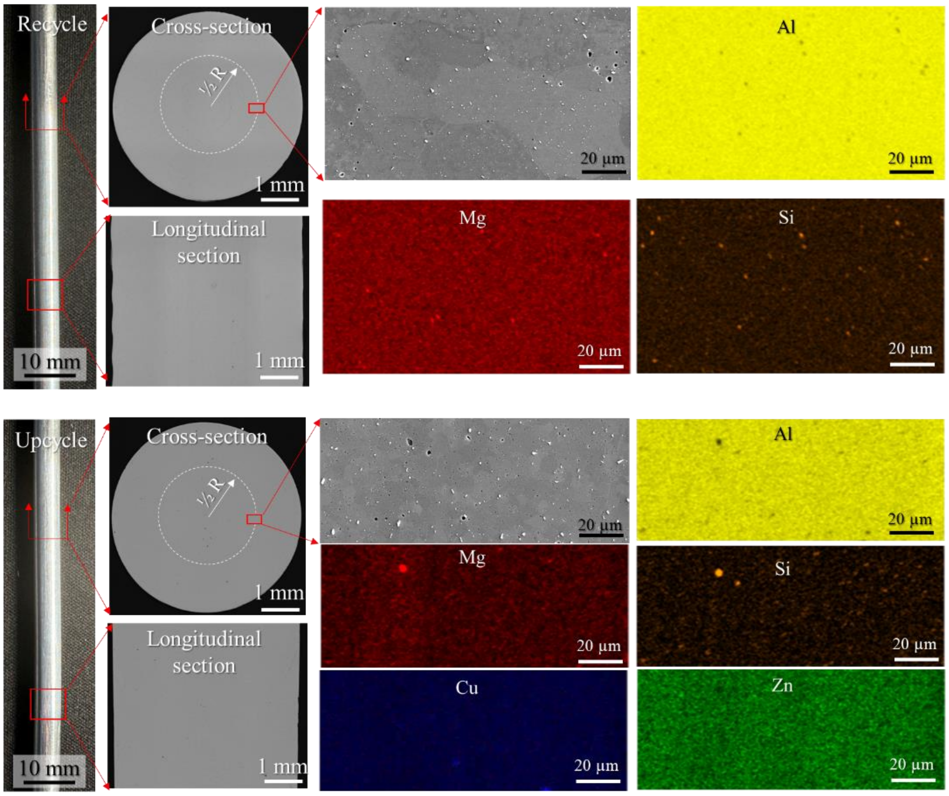

Supplementary Figure 1. Optical microscopy images of a cross-section and longitudinal section of recycled and upcycled specimens, and SEM-EDS map analysis on the selected region from the cross-sections (at half radius).

Supplementary Note 2 Hardness of the recycled and upcycled rod

The hardness conducted on the cross-section of recycled and upcycled rods is displayed in Fig. 2. It shows that hardness is uniform for both conditions. The average hardness for the recycled rod is 43 HV, while the average hardness for the upcycled rod is 116 HV.

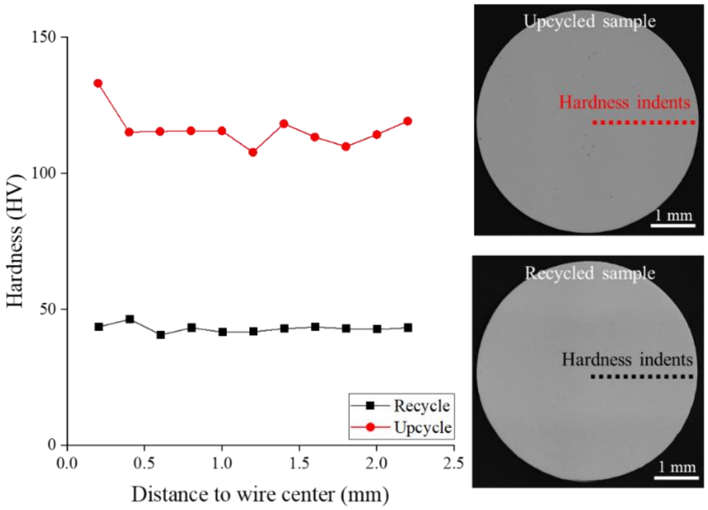

Supplementary Figure 2. Hardness distribution on the cross-section of recycled and upcycled specimens.

The hardness maps for extruded products in the as-upcycled condition and the upcycled+T6 condition are displayed in Fig. 3a and 3b, repectively.

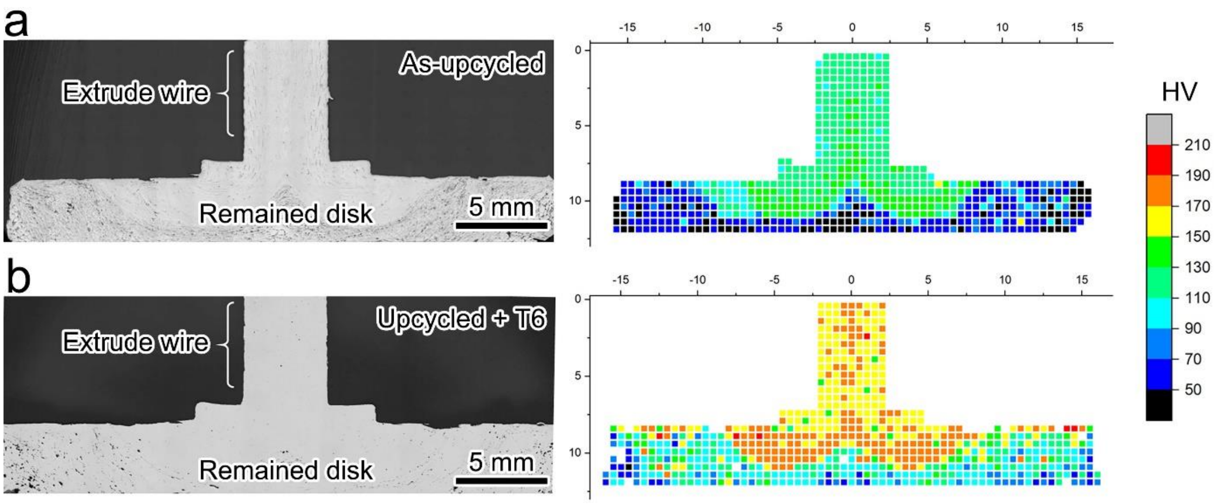

Supplementary Figure 3. Optical image displaying the remained disk and extruded wire and hardness map for **a**. the as-upcycled condition and **b.** the upcycled + T6 condition.

Supplementary Note 3 Precipitate evolution during upcycling

High-resolution transmission electron microscopy shows that the newly formed phases are identified as amorphous Mg(ZnCu), as shown in selected regions A, B, and C in Fig. 4. The crystallized η phase/$Mg(CuZn)_2$ formed within the amorphous phase, as indicated by the yellow cycles.

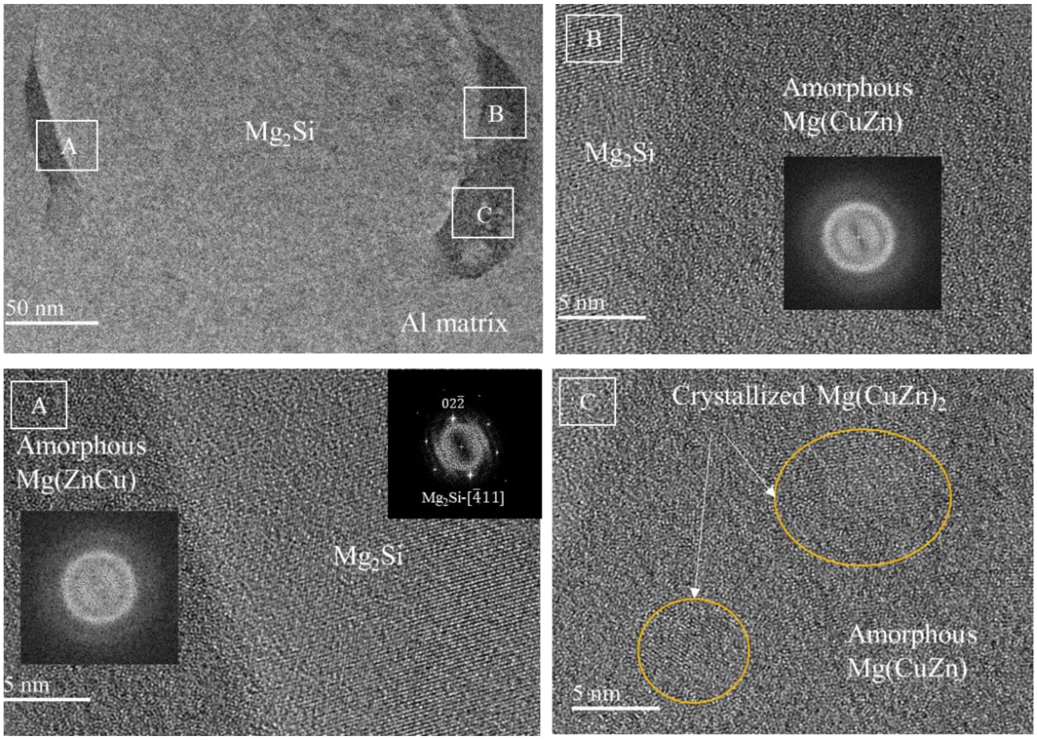

Supplementary Figure 4. TEM analysis shows the reaction product next to pre-existing Mg_2Si particles during upcycling.

Fig. 5a shows the frequency distribution of the second nearest neighbor (2NN) distance of Zn, Mg, and Cu atoms. The peak frequencies of experimental Zn-Zn, Mg-Mg, and Zn-Mg-Cu shift to smaller distances compared to the random ones, indicating a stronger clustering tendency between Zn, Mg, and Cu than Cu-Cu. The Zn/Mg ratio as a function of the cluster size, i.e., number of solute atoms (Fig. 5b), shows that the Zn-Mg-Cu clusters with a minimum size of N_{min} = 50 atoms have an average Zn/Mg ratio of ~1.4, which agrees with the reported Guinier-Preston (GP) II zones in Al-Zn-Mg-Cu alloys [SR1-3]. The number density of the GP zones is ~1.6×10^{24} m^{-3}, which is one order magnitude larger than typical values for Al-Mg-Si-Cu 6061-T6 alloys [SR4,5].

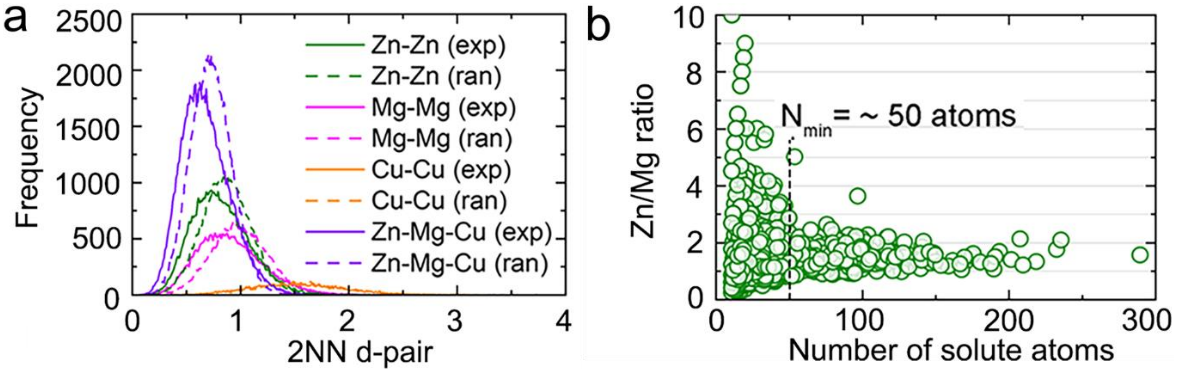

Supplementary Figure 5. a. Experimental and random frequency distribution of the 2NN distance between solute atoms. **b.** Zn/Mg ratio plotted against the number of solute atoms obtained from a selected volume with dimensions of 25 × 25 × 150 nm^3.

Fig. 6a shows the 3D atom maps of O, AlO, and Al_2O ions from the atomic probe tomography (APT) mass-to-charge spectra of the upcycled sample. The O^+, AlO^{2+}, AlO^+, and Al_2O^+ peaks are clearly detected in the correponding mass-to-charge spectrum (Fig. 6b). Fig. 6c shows the 1D compositional profiles along the arrow direction of a selected volume of 25 × 25 × 100 nm^3 in the inset. All oxide species detected are uniformly distributed in the matrix with concentrations less than 0.1 at.%.

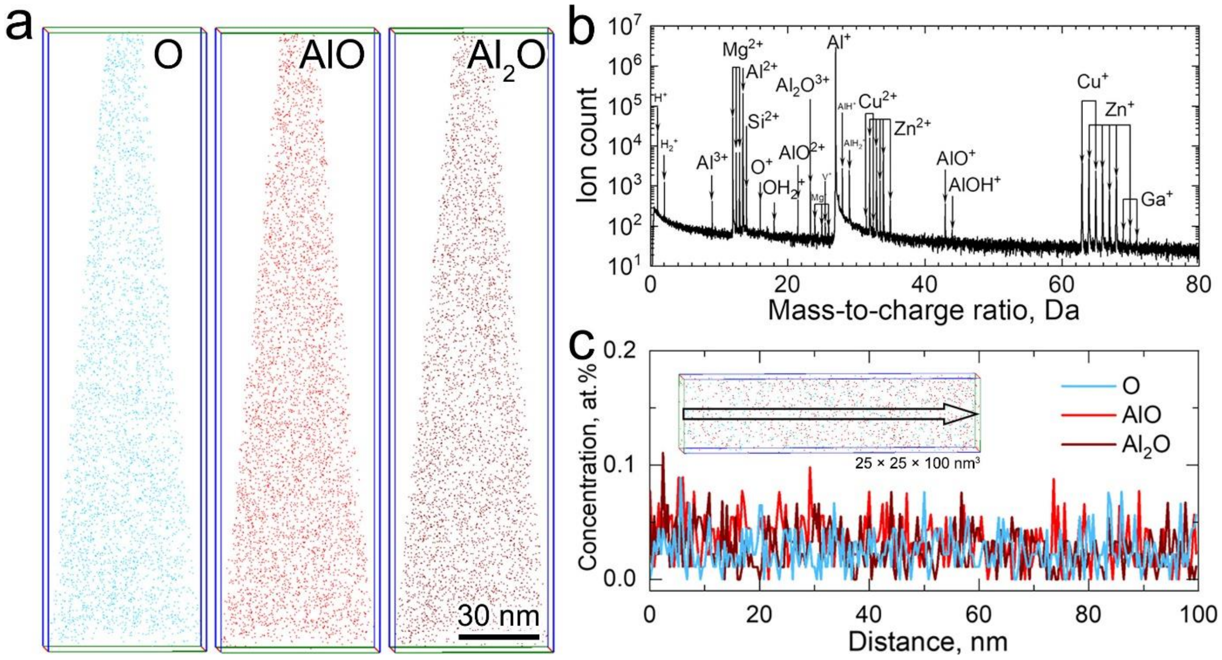

Supplementary Figure 6. APT results from the upcycled sample. **a.** 3D atom maps of O, AlO, and Al_2O, **b.** mass-to-charge spectrum, and **c.** 1-D compositional profiles along the arrow direction of a selected volume of 25 × 25 × 100 nm^3 in the inset.

Supplementary Note 4 Grain boundary strengthening

$$\Delta\sigma_{gb} = \frac{k_y}{\sqrt{d}} \quad (1)$$

where $\Delta\sigma_{gb}$ represents the strength contribution from grain boundaries, k_y represents the Hall-Petch coefficient and is ~0.12 MPa·m$^{1/2}$ for the 7075 Al alloy (upcycled wire) [SR6] and ~0.086 MPa·m$^{1/2}$ for the 6063 Al alloy (recycled wire) [SR7], and d represents the average grain diameter. The calculated strength contributions from grain boundaries are tabulated in Table 1.

Supplementary Table 1. Grain boundary strengthening contributions based on grain size.

Condition	Grain diameter, average (um)	Hall-Petch coefficient (MPa·m$^{1/2}$)	$\Delta\sigma_{gb}$ (MPa)
Upcycled	7.7	0.12	43.2
Recycled	43.1	0.086	13.1

Supplementary Note 5 Solid solution strengthening

Solid solution strengthening depends on solute type and concentration, which indicates the alloying element and concentration in recycled and upcycled wires. In the current work, practical correlations between the solute concentration and yield strength provided in previous studies [SR6] were applied and are listed in Table 2. Based on EDS results obtained from both recycled and upcycled Al alloys, $\Delta\sigma_{SS}$ was also calculated.

Supplementary Table 2. Solid solution strengthening contributions based on solute concentrations.

Condition	Solid solution strengthening	
Upcycled	Mg content (wt%)	1.2
	Contribution from Mg (MPa/wt%)	18.6
	Zn content (wt%)	5.26
	Contribution from Zn (MPa/wt%)	2.9
	Cu content (wt%)	0.67
	Contribution from Cu (MPa/wt%)	13.8
	Overall $\Delta\sigma_{ss}$ (MPa)	46.8
Recycled	Mg content (wt%)	0.4
	Contribution from Mg (MPa/wt%)	18.6
	Overall $\Delta\sigma_{ss}$ (MPa)	7.4

Supplementary Note 6 Precipitate strengthening

The strength of precipitation is controlled by either the Orowan dislocation bypassing or dislocation shearing mechanism. Note that the strength attributed to GP zones (coherent to the Al matrix) is expected to arise from the dislocation shearing mechanism rather than the Orowan dislocation bypassing mechanism. Nonetheless, a prior investigation [SR6] revealed that $\Delta\sigma_{Orowan}$ (dislocation bypassing) is smaller than $\Delta\sigma_{CS}$ (coherency strength) for GP zones. Consequently, the Orowan dislocation bypassing mechanism is established as the operative mechanism for GP zones in the present study.

Orowan equation: $$\Delta\sigma_{Orowan} = \mathrm{M}\frac{0.4\mathrm{Gb}}{\pi\sqrt{1-v}}\frac{\ln(2\sqrt{2/3}\mathrm{r/b})}{\lambda_p} \quad (2)$$

$$\lambda_p = \mathrm{r}\left(\sqrt{\frac{2\pi}{3f}} - 1.63\right) \quad (3)$$

where M represents the mean orientation factor and is ~3.06 for the fcc polycrystalline matrix, G represents the shear modulus and is 25.8 for the 6063 Al alloy and 26.9 for the 7075 Al alloy, b represents the

magnitude of the Burger's vector and is ~0.286 for fcc metals, $\Delta\sigma_{Orowan}$ represents the strength contribution from precipitates, ν represents Poisson's ratio and is ~0.33 for Al, r represents the mean radius of the precipitates, and λ_p represents the space between precipitates. Based on the microstructural characterization results presented in the main text, the variation in precipitate strengthening contributions between recycled and upcycled specimens comes from the atom clusters of GP zones and η' precipitates. According to the analysis of the upcycled specimen presented in the main text (Fig. 4), the presence of η' precipitates is deemed negligible for strength calculations. Consequently, this section exclusively considers the strength contribution stemming from GP zones. The APT analysis furnishes information on the average size and volume fraction of GP zones in the upcycled specimen. The resulting strength contributions from GP zones in the upcycled specimen are detailed in Table 3.

Supplementary Table 3. Precipitate size, volume fraction, and strengthening contribution in the upcycled specimen.

Condition	Average size of GP zone (nm)	Volume fraction (%)	$\Delta\sigma_{orowan}$ (MPa)
Upcycled	1.22	0.077	115.9

Supplementary Note 7 Dislocation strengthening

The dislocation densities in the upcycled and recycled specimens were quantitatively determined by the simplified breadth method via X-ray diffraction [SR8,9]. The domain size D is calculated by equation (3) based on the wavelength λ, the Cauchy component of the integral breadth β_C, and the Bragg angle θ. The strain ε is calculated by equation (4) based on the Gauss component of the integral breadth β_G and the Bragg angle θ. The dislocation density is calculated by equation (5) based on D, ε, and Burger's vector b [SR10].

$$D = \frac{\lambda}{\beta_C \times \cos\theta} \quad (3)$$

$$\varepsilon = \frac{\beta_G}{\tan\theta} \quad (4)$$

$$\rho = \frac{2\sqrt{3} \times \varepsilon}{\mathrm{D} \times \mathrm{b}} \quad (5)$$

The strength contribution from dislocations can be obtained from the following equation (6)

$$\Delta\sigma_d = M \times \alpha \times G \times b \times \sqrt{\rho} \quad (6)$$

where M = 3.06 is the mean orientation factor (~3.06 for the fcc polycrystalline matrix), α = 0.2 is a constant, and G is the shear modulus at 26.9 GPa for the Al 7075 alloy. The calculated strength from dislocations is tabulated in Table 4.

Supplementary Table 4. Strengthening contribution from dislocations in the upcycled and recycled specimens.

Condition	Dislocation strengthening		Condition	Dislocation strengthening	
Upcycled	β_c	0.047	Recycled	β_c	0.08
	β_G	0.308		β_G	0.23
	D	320.03		D	188.08
	ε	0.00301		ε	0.00225
	b (nm)	0.286		b (nm)	0.286
	λ (nm)	0.228973		λ (nm)	0.228973
	θ (°)	58.57		θ (°)	586.4
	ρ (×10^14/m²)	1.14101		ρ (×10^14/m²)	1.45188
	$\Delta\sigma_d$ (MPa)	52.3		$\Delta\sigma_d$ (MPa)	56.7

Supplementary Note 8. Size and morphology of Cu and Zn powders

Both Cu and Zn powders are ~325 mesh, indicating particle diameters of less than 44 μm. The morphology and composition of the Cu and Zn powders are presented through SEM images and EDS analysis, as shown in Fig. 7.

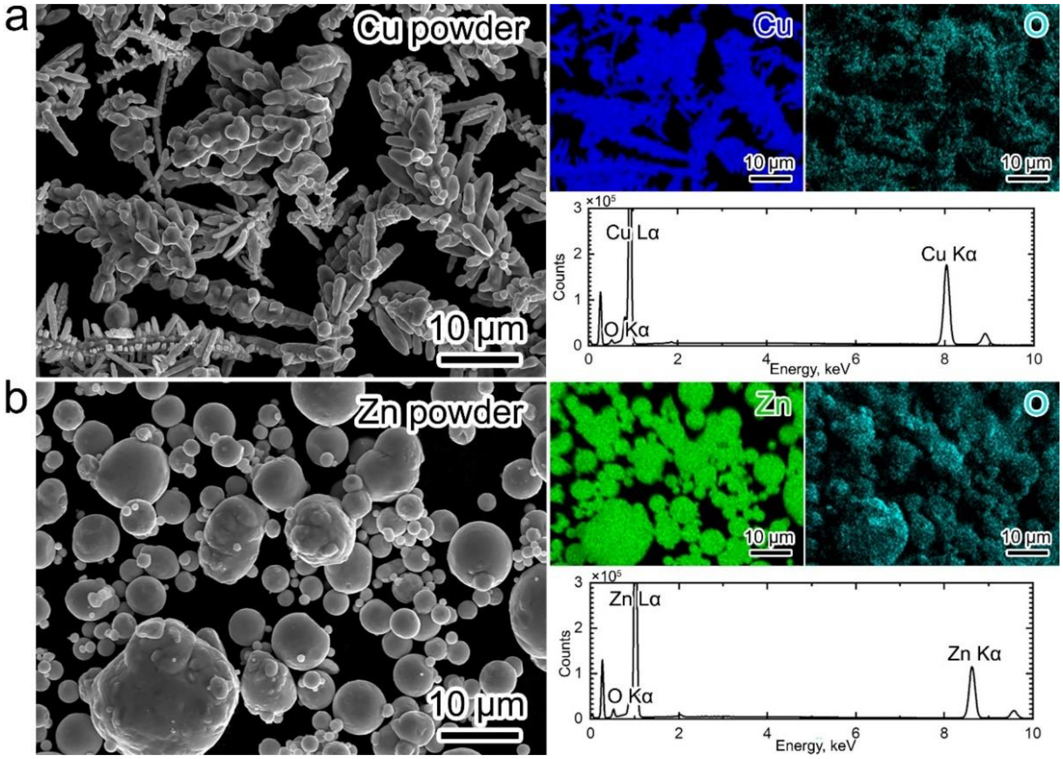

Supplementary Figure 7. SEM and EDS analysis of **a**. Cu powder and **b.** Zn powder.

Supplementary References

[SR1] Sha, G. and Cerezo, A., 2004. Early-stage precipitation in Al–Zn–Mg–Cu alloy (7050). Acta materialia, 52(15), pp.4503-4516.
[SR2] Liu, J., Hu, R., Zheng, J., Zhang, Y., Ding, Z., Liu, W., Zhu, Y. and Sha, G., 2020. Formation of solute nanostructures in an Al–Zn–Mg alloy during long-term natural aging. Journal of Alloys and Compounds, 821, p.153572.
[SR3] Lee, S.H., Jung, J.G., Baik, S.I., Seidman, D.N., Kim, M.S., Lee, Y.K. and Euh, K., 2021. Precipitation strengthening in naturally aged Al–Zn–Mg–Cu alloy. Materials Science and Engineering: A, 803, p.140719.
[SR4] Buchanan, K., Colas, K., Ribis, J., Lopez, A. and Garnier, J., 2017. Analysis of the metastable precipitates in peak-hardness aged Al-Mg-Si (-Cu) alloys with differing Si contents. Acta Materialia, 132, pp.209-221.
[SR5] Lervik, A., Thronsen, E., Friis, J., Marioara, C.D., Wenner, S., Bendo, A., Matsuda, K., Holmestad, R. and Andersen, S.J., 2021. Atomic structure of solute clusters in Al–Zn–Mg alloys. Acta Materialia, 205, p.116574.
[SR6] Ma, K., Wen, H., Hu, T., Topping, T.D., Isheim, D., Seidman, D.N., Lavernia, E.J. and Schoenung, J.M., 2014. Mechanical behavior and strengthening mechanisms in ultrafine grain precipitation-strengthened aluminum alloy. Acta Materialia, 62, pp.141-155.
[SR7] Bui, Q.H., Pham, X.T. and Fafard, M., 2013. Modelling of microstructure effects on the mechanical behavior of aluminium tubes drawn with different reduction areas. International Journal of Plasticity, 50, pp.127-145.
[SR8] De Keijser, Th H., et al. "Use of the Voigt function in a single-line method for the analysis of X-ray diffraction line broadening." Journal of Applied Crystallography 15.3 (1982): 308-314
[SR9] Langford, J. Il. "A rapid method for analysing the breadths of diffraction and spectral lines using the Voigt function." Journal of Applied Crystallography 11.1 (1978): 10-14.
[SR10] Dasharath, S.M. and Mula, S., 2016. Microstructural evolution and mechanical properties of low SFE Cu-Al alloys processed by cryorolling followed by short-annealing. Materials & Design, 99, pp.552-564.
